# Antidyskinetic Effects of MEK Inhibitor Are Associated with Multiple Neurochemical Alterations in the Striatum of Hemiparkinsonian Rats

**DOI:** 10.3389/fnins.2017.00112

**Published:** 2017-03-09

**Authors:** Guiqin Chen, Shuke Nie, Chao Han, Kai Ma, Yan Xu, Zhentao Zhang, Stella M. Papa, Xuebing Cao

**Affiliations:** ^1^Department of Neurology, Union Hospital, Tongji Medical College, Huazhong University of Science and TechnologyWuhan, China; ^2^Department of Neurology, Renmin Hospital of Wuhan UniversityWuhan, China; ^3^Department of Neurology, Yerkes National Primate Research Center, Emory University School of MedicineAtlanta, GA, USA

**Keywords:** Parkinson's disease, L-DOPA-induced dyskinesia, p-ERK1/2, ΔFosB, TH, ARNT, PD98059, microarray

## Abstract

L-DOPA-induced dyskinesia (LID) represents one of the major problems of the long-term therapy of patients with Parkinson's disease (PD). Although, the pathophysiologic mechanisms underlying LID are not completely understood, activation of the extracellular signal regulated kinase (ERK) is recognized to play a key role. ERK is phosphorylated by mitogen-activated protein kinase kinase (MEK), and thus MEK inhibitor can prevent ERK activation. Here the effect of the MEK inhibitor PD98059 on LID and the associated molecular changes were examined. Rats with unilateral 6-OHDA lesions of the nigrostriatal pathway received daily L-DOPA treatment for 3 weeks, and abnormal involuntary movements (AIMs) were assessed every other day. PD98059 was injected in the lateral ventricle daily for 12 days starting from day 10 of L-DOPA treatment. Striatal molecular markers of LID were analyzed together with gene regulation using microarray. The administration of PD98059 significantly reduced AIMs. In addition, ERK activation and other associated molecular changes including ΔFosB were reversed in rats treated with the MEK inhibitor. PD98059 induced significant up-regulation of 418 transcripts and down-regulation of 378 transcripts in the striatum. Tyrosine hydroxylase (*Th*) and aryl hydrocarbon receptor nuclear translocator (*Arnt*) genes were down-regulated in lesioned animals and up-regulated in L-DOPA-treated animals. Analysis of protein levels showed that PD98059 reduced the striatal TH. These results support the association of p-ERK1/2, ΔFosB, p-H3 to the regulation of TH and ARNT in the mechanisms of LID, and pinpoint other gene regulatory changes, thus providing clues for identifying new targets for LID therapy.

## Introduction

L-DOPA-induced dyskinesia (LID) that are associated with chronic dopamine replacement therapy still represent one of the major problems in the management of patients with Parkinson's disease. Accumulating evidence manifests that overly sensitized dopamine D1 receptor transmission and its downstream signaling pathway play a key role in the mechanisms of LID (Gerfen et al., 2002; Westin et al., 2007; Darmopil et al., 2009; Feyder et al., 2011). Several postsynaptic signaling molecules are involved in this pathway, including phospho-Thr34-DARPP-32 (p-DARPP32), phosphorylated extracellular signal-regulated kinase 1/2 (p-ERK1/2), ΔFosB, and phospho-Ser10-histone H3 (p-H3) (Hakansson et al., 2004; Pavon et al., 2006; Santini et al., 2007; Cao et al., 2010; Du et al., 2015; Potts et al., 2015). In animal models of PD, striatal overactivation of these neurochemicals characterizes the molecular profile of the supersensitive response underlying dyskinetic behaviors. P-ERK1/2 and dopamine D1 receptor, are implicated in various forms of synaptic plasticity, particularly in late long-term potentiation (LTP) and thereby in cocaine addiction, learning and memory (Kelleher et al., 2004a,b; Granado et al., 2008; Borkar et al., 2013; Cahill et al., 2014; Suarez et al., 2014). The dopamine D1 receptor signaling pathway is associated with overactivation of ERK1/2 in rodent models of LID (Westin et al., 2007; Darmopil et al., 2009; Feyder et al., 2011). The phosphorylation level of ERK1/2 can be increased by D1 agonists (and decreased by D1 antagonists) in the striatum of rats with 6-hydroxydopamine (6-OHDA) lesions (Gerfen et al., 2002; Santini et al., 2009a) and even in intact animals (Gangarossa et al., 2013). ERK1/2 was also found to be the downstream signaling of the cAMP/PKA/DARPP-32 pathway in animal models of LID, although studies have been inconsistent (Santini et al., 2007; Dupre, 2008; Gerfen et al., 2008; Lebel et al., 2010). In addition, ERK1/2 appears to activate the mammalian target of rapamycin complex 1 (mTORC1) (Roux et al., 2007; Carriere et al., 2008) that is a critical regulator of mRNA translation especially in relation to long-lasting synaptic plasticity and memory (Costa-Mattioli et al., 2009). Persistent activation of mTORC1 mediated by ERK1/2-D1 receptor stimulation has been found in the striatum of a mouse LID model (Santini et al., 2009b). Rapamycin and CCI-779, the inhibitor of mTOR, modify mTORC1 targets and can reduce abnormal involuntary movements (AIMs) in rodents (Santini et al., 2009b; Decressac and Björklund, 2013).

Other established molecular hallmark of LID is ΔFosB, a truncated isoform of FosB, that is a chronic transcription factor. ΔFosB possesses unique stability properties compared with all other Fos family members (Andersson et al., 1999, 2003; Cenci, 2002; Cao et al., 2010). Chronic L-DOPA treatment induces FosB/ΔFosB accumulation in the striatum that may result not only in dyskinesia but also an insensitive response to L-DOPA (Engeln et al., 2016). Furthermore, alternative inactivation of FosB-ΔFosB-expressing neurons in the striatum attenuates LID (Doo et al., 2014; Engeln et al., 2016). Another important marker is p-H3 whose posttranslational modification is induced by L-DOPA (Santini et al., 2007, 2009a). P-H3 is co-expressed with dynorphin in striatal neurons, and thus, its changes may associate with the transcriptional alterations underlying LID (Darmopil et al., 2009).

We aimed at reducing the phosphorylation of ERK1/2 to examine its role in the regulation of other biomarkers of LID and associated transcriptional changes. We used the MEK inhibitor PD98059 and whole transcriptome analysis using microarray analysis in rats with unilateral 6-OHDA lesions and chronic exposure to L-DOPA treatment. In line with previous work (Santini et al., 2007), PD98059 prevented the phosphorylation of ERK1/2 and histone H3, and reduced the abnormal accumulation of ΔFosB. Also, the inhibitor showed a powerful effect of counteracting AIMs. Importantly, numerous genes were regulated in association with AIMs. We focused on the function of the following genes, tyrosine hydroxylase (Th) and aryl hydrocarbon receptor nuclear translocator (*Arnt*), which regulates *Th* (Teh et al., 2007). The administration of PD98059 neutralized the L-DOPA-induced changes in Arnt and Th levels. These results provide evidence for a significant interplay between LID biomarkers (p-ERK1/2, ΔFosB, p-H3) and the regulation of striatal *Th* and *Arnt* genes in the pathophysiology of LID development. The analysis of the transcriptome may also provide clues for identifying further genes that are involved in LID mechanisms.

## Materials and methods

### Animals

Adult, male *Sprague–Dawley* rats (Beijing HFK Bioscience Co., Ltd., China) weighing 230–250 g (7 weeks old) were housed with free access to food and water, 12 h light/dark cycle, constant temperature and humidity. Animal use and care were conformed to the Guidelines of Laboratory Animals Ethics of Tongji Medical College, Huazhong University of Science and Technology. The protocol was approved by the Ethics Committee of Huazhong University of Science and Technology.

### Drugs

6-OHDA (2 μg/μl) and apomorphine hydrochloride (0.1 μg/μl) were dissolved in saline with 0.02% ascorbic acid, and L-DOPA methyl ester and benserazide (12 and 6 mg/kg) were dissolved in saline immediately before use (Sigma-Aldrich). PD98059 (Calbiochem) was dissolved in 20% DMSO, 10% Tween80, and diluted to 0.4 μg/μl with saline (Miller and Marshall, 2005). Both isoflurane and pentobarbital were purchased from Sigma-Aldrich.

### 6-OHDA lesion and cannula implantation

**Figure 1:**
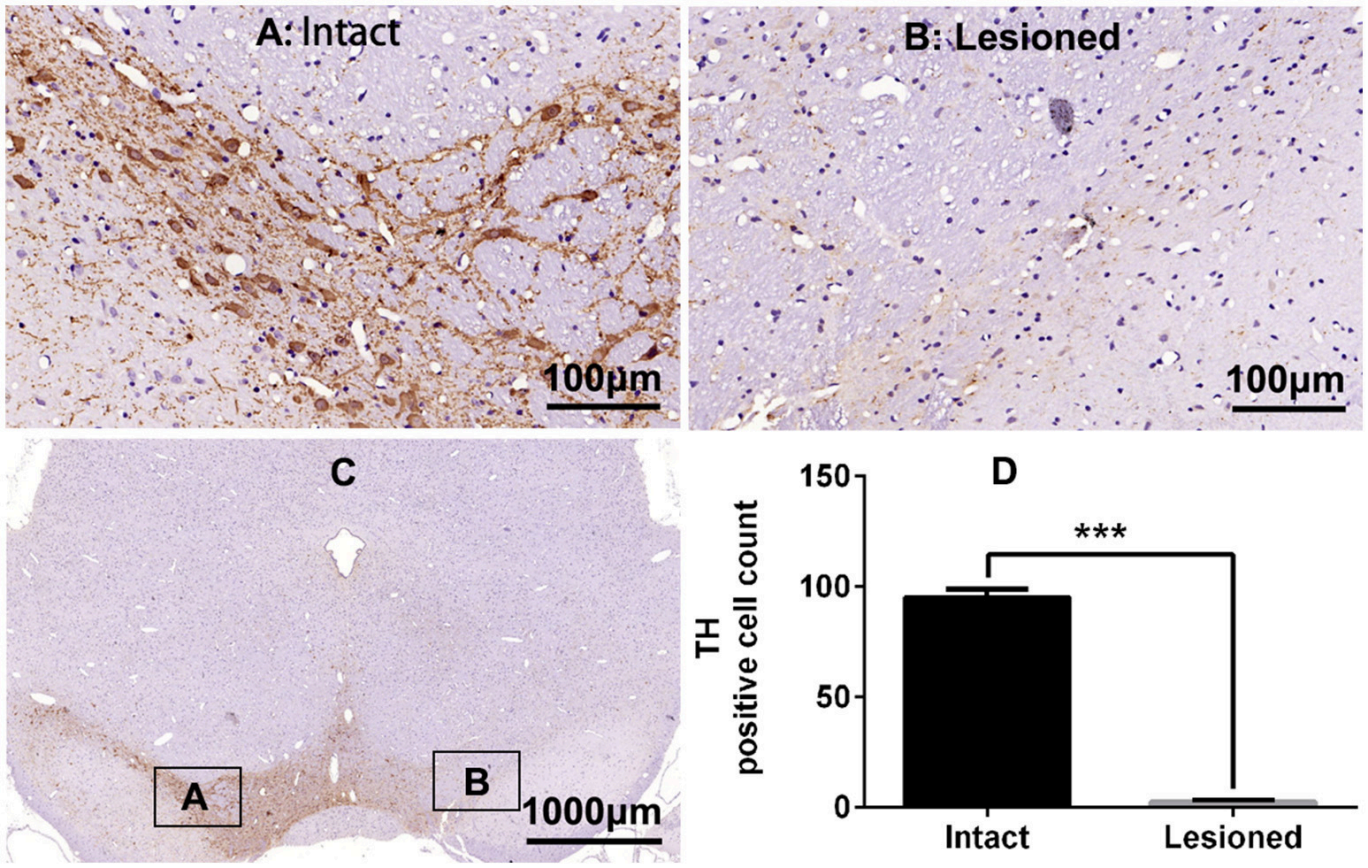
**Tyrosine hydroxylase (TH) immunostaining in substantia nigra (SN) in the rat 6-OHDA lesion model. (A,B,D)** Analysis of differences in TH positive cell counts in SN of the intact and lesioned side. ^***^p < 0.001 (*t*-test, *N* = 4/group). Scale bar is 100 μm. Error bars represent SEM. **(C)** Integral field-vision of the SN with TH immunostaining. The boxes indicate the SN in which positive neurons are counted (an area of 0.2 mm^2^).

Rats were deeply anesthetized with isoflurane (induction 3%, maintenance 1.5%) in oxygen and mounted on the stereotaxic frame. A temperature controller system was used to maintain body temperature at 37°C. 6-OHDA (2 μg/μl, 4 μl) was injected into the right medial forebrain bundle through a 10-μl microsyringe at a rate of 0.5 μl/min at the following stereotactic coordinates: relative to bregma, −4.4 mm anterior (A), +1.5 mm lateral (R), and 7.8 mm deepness (D) (Lindgren et al., 2009). In 33 rats, a guide cannula connected to a microinjection system (RWD Life Science Co., Ltd) was implanted to the right lateral ventricle (A −0.7 mm, R +1.5 mm, D 4.0 mm) according to atlas of Paxinos and Watson (2005) and secured to the skull with dental cement. The efficacy of the dopaminergic lesion was tested by measuring contralateral turning behavior with an acute subthreshold dose of apomorphine (0.05 mg/kg s.c.) 2 weeks post-surgery. Only rats exhibiting more than 200 turns contralateral to the lesion side in 30 min were considered to be compatible with the model of full lesion, and were chosen for further study (Boldry et al., 1995). TH staining revealed also a complete lesion of substantia nigra (SN) ipsilateral to the lesion (see Figure 1).

### Drugs treatment and behavioral assessment

**Figure 2:**
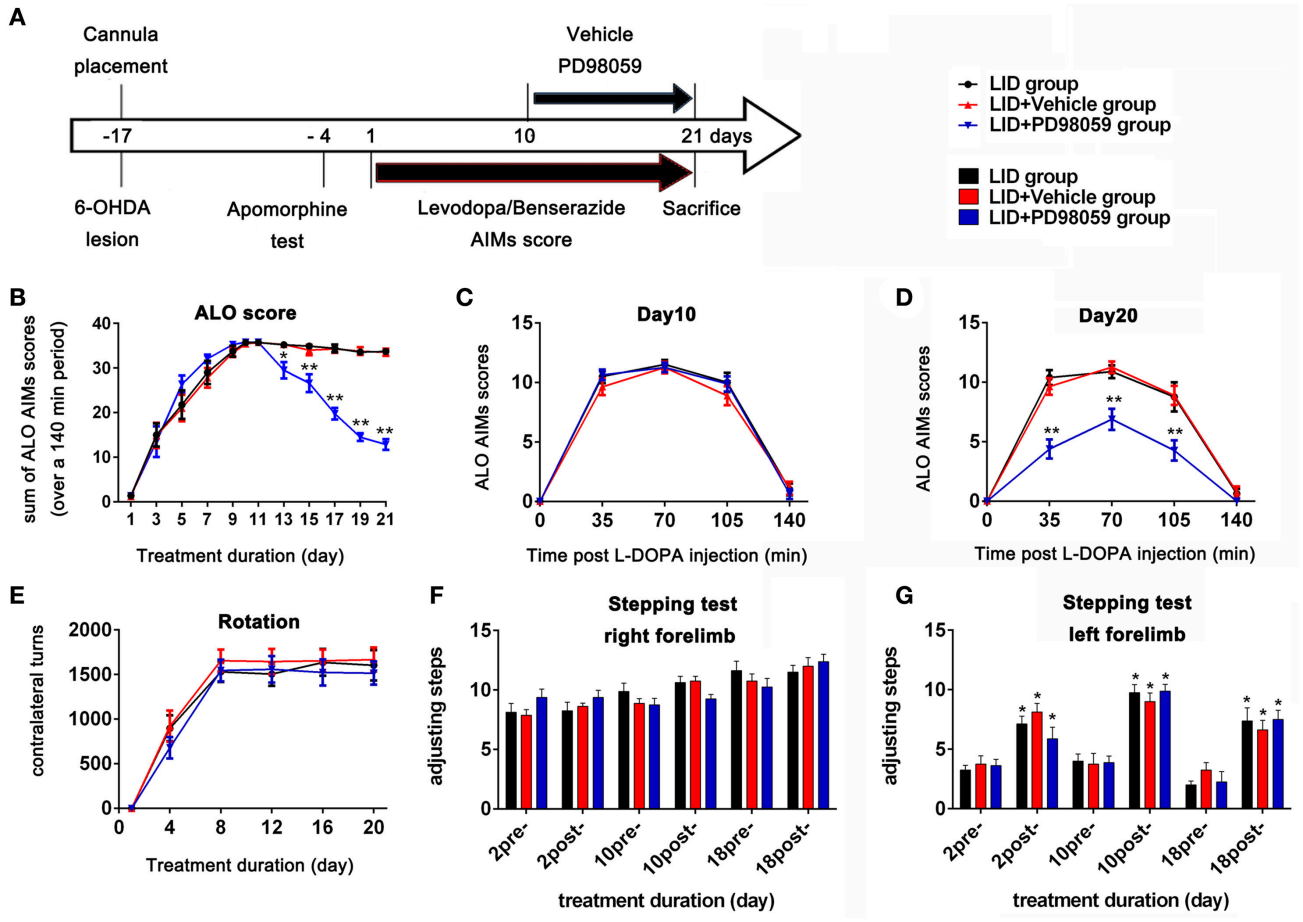
**Experimental design and behavior changes induced by different treatment. (A)** Timeline of experiments Studies began with cannula placement into the lateral ventricle and 6-OHDA lesions of MFB followed by apomorphine test identifying rats with complete lesions. The bottom arrow shows the timeline for all groups of chronic treatment with L-DOPA methyl ester (12 mg/Kg) plus benserazide (6 mg/Kg) once daily. Behavioral assessment was done every other day. The upper arrow shows the daily lateral ventricle infusion with vehicle or PD98059 half hour before L-DOPA injection. All animals were sacrificed on day 21 for further studies (*N* = 14/group). **(B–E)** Effect of PD98059 on AIMs in 6-OHDA-lesioned rats **(B)**. ALO (total of axial, limb, and orofacial) AIMs scores that were obtained every 35 min over 140 min following the L-DOPA injection every other day. **(C,D)** Total ALO AIMs scores within 140 min sessions following drug administration on day 10 **(C)** and 20 **(D)**. **(E)** Contralateral turns that were counted on days 1, 4, 8, 12, 16, and 20. **(F,G)** Stepping tests scores 15 min before (pre-) and after (post-) L-DOPA administration on day 2, 10, and 18. ^*^p < 0.05, ^**^p < 0.01 in **(B–E)**, LID + PD98059 vs. LID + Vehicle, ^*^p < 0.05 in **(F,G)**, post-L-DOPA vs. pre-L-DOPA administration (one-way ANOVAs followed by Tukey HSD and LSD *post-hoc* test). Error bars represent SEM.

Three days after apomorphine screening (Figure 2A), 45 successful hemiparkinsonian rats received intraperitoneal injections of L-DOPA plus benserazide (12/6 mg/kg) once daily. L-DOPA-induced AIMs were recorded every other day. Rats were observed for 1 min every 35 min intervals for a total of 140 min following L-DOPA treatment. AIMs were evaluated using the validated AIMs scale. Orofacial, limb, and axial dyskinesia were graded from score 0 to 4: 0 = absent; 1 = occasional, present during less than half min; 2 = frequent, present during more than half min; 3 = continuous but interrupted by strong sensory distraction; 4 = continuous, not interrupted by strong sensory distraction (Winkler et al., 2002; Lundblad et al., 2004). The total of axial, limb and orofacial dyskinesia was also named ALO dyskinesia and the maximum ALO dyskinesia score in each session was 48. Rotation (contralateral turns) test was performed as before (Breger et al., 2013) at day 1, 4, 12, 16, and 20, and only turns of completed 360° were counted.

From day 10, 42 rats with complete dyskinesia (ALO AIMs score = 12 at 70 min after L-DOPA) were divided into 3 groups: LID group, LID + PD98059 group, and LID + vehicle group. There was cannula implantation for each rat in the latter two groups. A volume of 3 μl PD98059 solution (0.4 μg/μl) or vehicle was infused into the right lateral ventricle over 6 min through the cannula connected to the microinjection system half hour before L-DOPA administration from day 10 to 21 (Figure 2A). The infusion needle was left in place for 2 min at the end of infusion for drug diffusion. Normal rats with sham operation and hemiparkinsonian rats were chosen as normal group and PD group, respectively. There were 14 rats in each group.

The stepping test was carried out 15 min before (pre-) and after (post-) L-DOPA treatment as previously described (Pinna et al., 2007, 2010) on days 2, 10, and 18. Rats were moved on the surface of the table for 0.7 m in 4 s by the experimenter. The number of adjusting steps of left and right forelimbs in the forward directions was counted.

All behavioral tests were performed by a blinded examiner.

### Tissue preparation

At day 21, 4 h after the last L-DOPA treatment, 4 rats of each group (normal, PD, LID, LID + Vehicle, and LID + PD98059) were anesthetized with an overdose of pentobarbital and then perfused transcardially with saline followed by 4% ice-cold paraformaldehyde (PFA) in phosphate buffer (pH 7.4). Brain tissues were removed, post-fixed with 4% PFA overnight and 25% sucrose for 24 h. Then the fixed samples were embedded in paraffin for immunohistochemistry and immunofluorescence. Another 10 rats of each group were sacrificed by decapitation. Brain tissues were rapidly removed, dissected for striatum on powdered dry ice. Striatum from 4 of the 10 rats were immediately stored at −80°C until protein extraction for Western blotting analysis, and 6 of the 10 were incubated in RNAlater (Qiagen) at 4°C overnight and then stored at −80°C for further Microarray analysis (3 rats) and real-time quantitative PCR (3 rats).

### Western blotting

Brain tissues were homogenized with glass homogenizers in ice-cold enhanced RIPA lysis buffer, containing 50 mM Tris (pH 7.4), 150 mM NaCl, 1% Triton X-100, 1% sodium deoxycholate, 0.1% SDS, 5 mM EDTA, 2 mM Na3VO4, 1 mM PMSF, 10 mM NaF, and a complete set of protease inhibitors (Roche, USA). Lysates were centrifuged at 12,000 g at 4°C for 15 min and the protein concentrations were determined by a BCA assay kit (Pierce, Rockford). The resulting supernatant was added with 1% SDS loading buffer, and boiled for 10 min. Equal amounts of protein (some 40 μg) of each sample were separated on 10% SDS–polyacrylamide gel electrophoresis (SDS-PAGE), transferred for 90 min onto polyvinylidene fluoride (PVDF) membranes (Millipore, USA), blocked for 1 h at 25°C in 5% non-fat powdered milk dissolved in Tris-buffered saline containing 0.1% Tween 20 (TBST), and immunoblotted overnight at 4°C with primary antibodies. The following primary antibodies were deployed: rabbit polyclonal antibody anti-ΔFosB (1:500; Cell Signaling Technology; #9890), rabbit monoclonal antibody against p44/42 MAPK (ERK1/2; 1:1,000; Cell Signaling Technology; #4695), rabbit monoclonal antibody against phospho-p44/42 MAPK (p-ERK1/2; Thr202/Tyr204; 1:1,000; Cell Signaling Technology; #4370). The membranes were washed in TBST, incubated with horseradish peroxidase-conjugated goat anti-rabbit secondary antibody (1:5,000; GeneTex: GTX213110-01), and visualized with an ECL detection kit (Thermo Scientific). The membranes were stripped and incubated with rabbit polyclonal antibody against β-actin (1:1,000; AntGene; ANT010) as a loading control. Bands intensities were analyzed quantitatively by Gel Pro Analyzer version 6.0 (Media Cybernetics, Bethesda, MD, USA). Densitometry was represented as relative optical density. All Western blots were repeated not less than three times.

### Immunohistochemistry and immunofluorescence

The paraffin-embedded brain tissues were sectioned at thickness of 4 μm. SN and striatum sections were mounted on glass slides, deparaffinized by xylene for 15 min (2 times), dehydrated in graded ethanol solutions, baked in the basic antigen retrieval buffer (pH = 6.0), and washed with phosphate buffer (pH 7.4) for 5 min (3 times). After washing, sections were blocked with 3% Bovine Serum Albumin for 30 min at room temperature (RT), then incubated with diluted primary antibody in a humidified chamber overnight at 4°C overnight. The following primary antibodies were used: rabbit polyclonal antibody against FosB (1:100, sc-48, Santa Cruz, detecting FosB-ΔFosB) for the immunohistochemistry staining in striatum, rabbit polyclonal antibody against TH (1:750, ab112, Abcam) for the immunohistochemistry staining in SN. Then all sections were washed with phosphate buffer (pH 7.4) for 5 min (3 times), subsequently incubated with biotinylated goat anti-rabbit IgG at 37°C for 50 min, washed again as above, incubated with Horseradish peroxidase labeled streptavidin fluid at 37°C for 30 min, washed, followed by DAB solutions for 5 min, washed, counterstained with Harris hematoxylin for 3 min, dehydrated in graded ethanol solutions, and eventually cover slipped. Images were collected through an Olympus camera connected to the microscope at the same light intensity, and analyzed using Image-Pro Plus software by an independent experimenter blinded to the sections by counting the number of positive cells in the SN or dorsal striatum of the lesioned side (relative to bregma, A −4.5 mm for SN and A 0.6 mm for striatum sections, *N* = 4 sections for counting). TH staining in SN was used to estimate the extent of the dopaminergic lesion (shown in Figure 1).

Immunofluorescence staining shared a same procedure with immunohistochemistry staining before secondary antibodies incubation. Primary antibodies used in the double staining contained rabbit polyclonal antibody against FosB-ΔFosB (1:100, sc-48, Santa Cruz, detecting FosB-ΔFosB) and mouse monoclonal against p-H3 (1:200, Ab14955, Abcam). Primary antibodies used in the single staining contained mouse monoclonal antibody against HIF-1β (1:200, ab2771, Abcam, detecting ARNT) and rabbit polyclonal antibody against TH (1:750, ab112, Abcam). After being washed, sections were incubated in dark with an appropriately diluted Alexa 488- or Cyanine 3-coupled secondary antibodies for 50 min followed by DAPI (4′, 6-diamidino-2-phenylindole) dyeing nucleus for 10 min. Images were collected using laser confocal microscopy marked with image manipulation software, and analyzed using Image-Pro Plus software by an independent experimenter blinded to the sections by counting the number of positive cells in the dorsal striatum of the lesioned side (relative to bregma, A 0.6 mm for striatum sections, *N* = 4 sections for counting).

### Microarray

Samples (striatum of lesioned side) were sent to Shanghai biotechnology Corporation for whole transcriptome analysis using microarray. Total mRNA was extracted using TRIZOL Reagent (Cat#15596-018, Life technologies, Carlsbad, CA, US) according to the manufacturer's instructions. RNA integrity was evaluated with the Agilent Bioanalyzer 2,100 and RNA 6,000 Nano/Pico Kit (Agilent Technologies, Santa Clara, CA, US). Qualified total RNA was further purified by RNeasy micro kit (Cat#74004, QIAGEN, GmBH, Germany) and RNase-Free DNase Set (Cat#79254, QIAGEN, GmBH, Germany). Concentrations of extracted RNA were assessed with the Nanodrop spectrophotometer (Nanodrop Technologies). A total of 100–150 ng RNA per sample was reverse transcribed to double stranded cDNA and then transcribed into cRNA using the Genechip WT Expression Kit (Affymetrix). Second cycle was carried out following generation of cRNA in order to transform the cRNA into single-strand cDNA. The cDNA was fragmented and the Genechip WT Terminal Labeling Kit (Affymetrix) was used to label the single-stranded DNA with biotin. Samples were hybridized to an Affymetrix Genechip Rat Gene 2.0 ST Array Platform. Array hybridization and wash was performed using GeneChip® Hybridization, Wash and Stain Kit (Cat#900720, Affymetrix, Santa Clara, CA, US) in Hybridization Oven 645 (Cat#00-0331-220V, Affymetrix, Santa Clara, CA, US), and Fluidics Station 450 (Cat#00-0079, Affymetrix, Santa Clara, CA, US) according to the manufacturer's instructions. Slides were scanned by GeneChip® Scanner 3000 (Cat#00-00212, Affymetrix, Santa Clara, CA, US).

### Real-time quantitative PCR

Total mRNA was extracted and reversely transcribed using the same method as in microarray. Quantification of mRNAs was performed by real-time PCR using Agilent-Stratagene Mx3000P Q-PCR System. The following primers (Invitrogen) were used: *Th* forward, 5′-GACATTGGACTTGCATCTCTG-3′, and *Th* reverse, 5′-GCTGGTAGGTTTGATCTTGGT-3′; *Arnt* forward, 5′-GAACCGAGAATGGCTGTGGATG-3′, and *Arnt* reverse, 5′-GCTGTGACCTCTGGATTGTGTTAG-3′; *FosB* forward, 5′-GTGAGAGATTTGCCAGGGTC-3′, and *FosB* reverse, 5′-GTGAGAGATTTGCCAGGGTC-3′; *beta-actin* forward, 5′-GGAGATTACTGCCCTGGCTCCTA-3′, and *beta-actin* reverse, 5′-GACTCATCGTACTCCTGCTTGCTG-3′. The SYBR Green *Premix Ex Taq*™ GC (Takara, RR420A) was employed. The PCR started with 94°C for 5 min, and then continued with 40 cycles of 10 s at 94°C, 20 s at 60°C, and 15 s at 72°C followed by 1 cycle of 30 s at 94°C, 30 s at 55°C, and 30 s at 94°C. Amplification plots and dissociation curves were obtained to analyze PCRs product and confirm amplification specificity. Expression levels of mRNA were determined using the ΔΔCT method. Each sample was tested in triplicate.

### Data analysis

The Expression Console software (Affymetrix, Santa Clara, CA, US) was used to format the raw microarray data. Data pre-processing, including background adjustment, log fold transformation and normalization was completed using the “exon level” option in the software. Moreover, the dataset was normalized using the Robust Multi Array Average (RMA) method to control the inter-array variability. Normalized signal intensities of probes which belong to one transcript of each sample were processed by median for further data analysis. Gene chip and RNA quality were assessed by examining total mRNA expression for each striatum. We have submitted the microarray data to the GEO repository. The GEO accession numbers is GSE93695.

R software was used to screen the differential gene expression transcripts (DETs) among these samples. The adjusted *P* < 0.05 and fold change (FC) ≥ 1.2 or ≤ 0.8333 were used as the cut-off criteria. After getting the DETs, we proceeded with the functional analysis, searching the function and signaling pathway of the genes. In this step, we used the known databases mainly, including DAVID Gene Ontology (http://david.abcc.ncifcrf.gov/). Considering the large amount and complex branch structure of Gene ontology (GO) biological processes, we used a significance threshold *P* < 0.05 for biological process terms.

The co-expression analysis starts by constructing a matrix of pairwise correlations between all pairs of transcripts across samples of LID + PD98059, LID + Vehicle and LID group. We built an unsigned co-expression network with all the 37,177 transcripts in microarray using the Weighted Gene Correlational Network Analysis (WGCNA) package [PMID: 19114008]. GO term enrichment tests were performed for individual gene co-expression modules compared to a background set of all genes expressed in these brain samples using the R packages GOstats (version 2.26.0), biomaRt version (2.14.0), AnnotationDbi (version 1.20.7), and org.Hs.eg.db (version 2.8.0).

To specialize LID + PD98059 from other treatment, unsupervised hierarchical clustering of the candidates in every trait module was performed by bootstrapping analysis using MeV software (http://www.tm4.org/). Bootstrapping analysis provides confidence values for the stability of each cluster derived by hierarchical clustering.

RT-qPCR data were expressed as fold changes in relative gene expression compared with the Normal group using beta-actin levels as an endogenous control. The significance level was set at *p* < 0.05. Data are presented as mean ± SEM.

### Statistics

Data were analyzed using one-way analysis of variance (ANOVA) followed by Tukey HSD or LSD *post-hoc* tests for multiple comparisons between groups, and Student's *t*-test for comparing TH positive neurons in the intact and lesioned side of SN. All statistical analyses were performed in SPSS 21.0 software. The significance level was set at *p* < 0.05. Data are presented as mean ± SEM for behavioral assessments, immunohistochemical count, and blot quantifications.

## Results

### MEK inhibitor attenuated LID

TH immunostaining was used to determine the DA neurons loss in SN following 6-OHDA lesions (Figure 1). DA neurons significantly decreased in the lesioned side (*t* = 23.432, *p* < 0.001 Figure 1D). Chronic L-DOPA administration (12 mg/kg, s.c., once daily for 21 days, Figure 2A) to rats with unilateral nigrostriatal 6-OHDA lesion led to the development of increasingly severe AIMs (axial, orofacial, limb AIMs) and rotation, all reaching a plateau after day 9 (Figure 2B, LID and LID + Vehicle groups). MEK inhibitor PD98059 (1.2 μg, injected into the right lateral ventricle 30 min before L-DOPA on days 10–21) clearly attenuated L-DOPA-induced AIMs after day 13 (Figure 2B). The total ALO (axial, limb and orofacial) scores of each group (LID, LID + Vehicle, LID + PD98059) were 34.40 ± 0.90, 34.30 ± 0.90, 19.80 ± 1.33 on day 17 [Figure 2B, *F*_(2, 39)_ = 62.247, *p* < 0.01], respectively. ALO scores at day 20 were decreased by PD98059 at all time points (35, 70, and 105 min after L-DOPA; Figures 2C,D). In contrast, rotation (contralateral turns) showed little difference among groups (Figure 2E).

The Stepping test showed that L-DOPA could significantly improve motor function of the contralateral forelimb in rats with unilateral nigrostriatal 6-OHDA-lesion (Figures 2F,G). PD98059 did not affect the stepping test scores (Figures 2F,G).

### Effects of MEK inhibitor on the striatal expression of p-ERK1/2, FosB-ΔFosB, and p-H3

**Figure 3:**
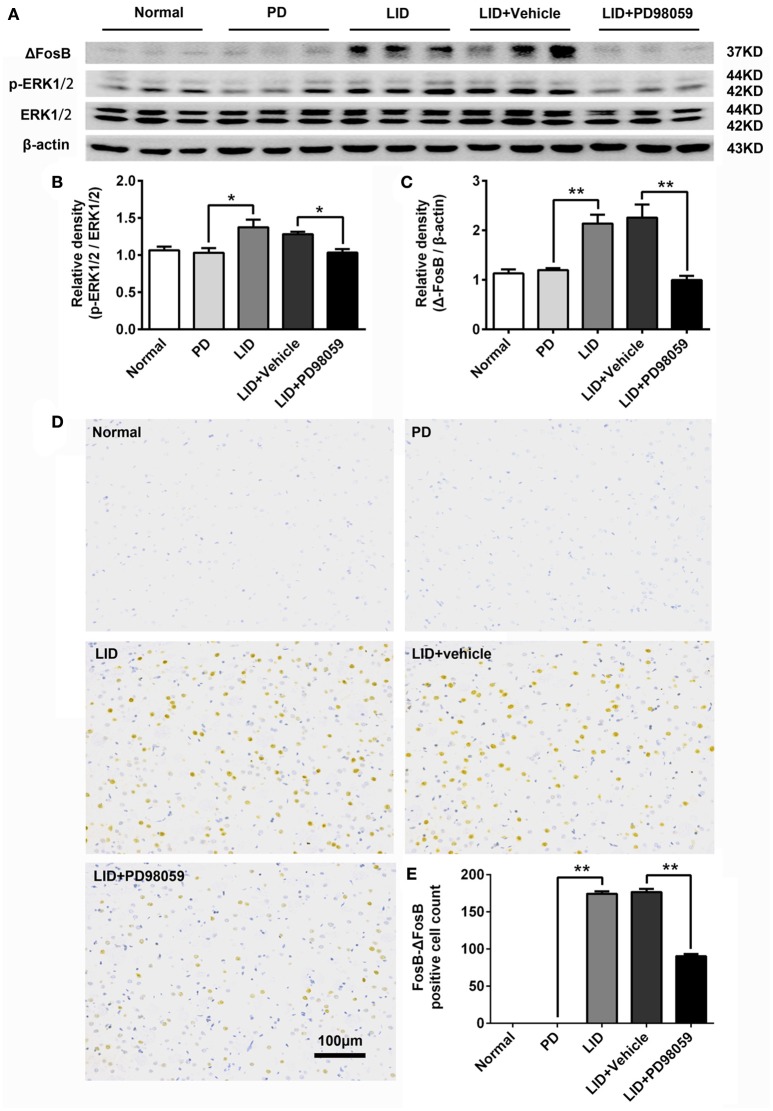
**PD98059 regulates the expression of p-ERK1/2 and FosB-ΔFosB in the striatum. (A)** Levels of ERK1/2, p-ERK1/2, and ΔFosB were relatively quantified in the DA-denervated striatum from each group of rats by Western blotting. **(B,C)** Analysis of differences in p-ERK1/2 vs. ERK1/2 and ΔFosB vs. β-actin in the lesioned striatum from each group. ^*^p < 0.05, ^**^p < 0.01 (one-way ANOVA followed by Tukey HSD and LSD *post-hoc* test, *N* = 4/group). Error bars represent SEM. **(D)** Immunohistochemical images of FosB-ΔFosB in dorsal striatum on the lesioned side from rats of each group (Normal, PD, LID, LID + Vehicle, LID + PD98059). Scale bar is 100 μm. **(E)** Analysis of differences in FosB-ΔFosB positive cells counts in each group. ^**^p < 0.01 (one-way ANOVA followed by Tukey HSD and LSD *post-hoc* test, *N* = 4/group). Error bars represent SEM.

**Figure 4:**
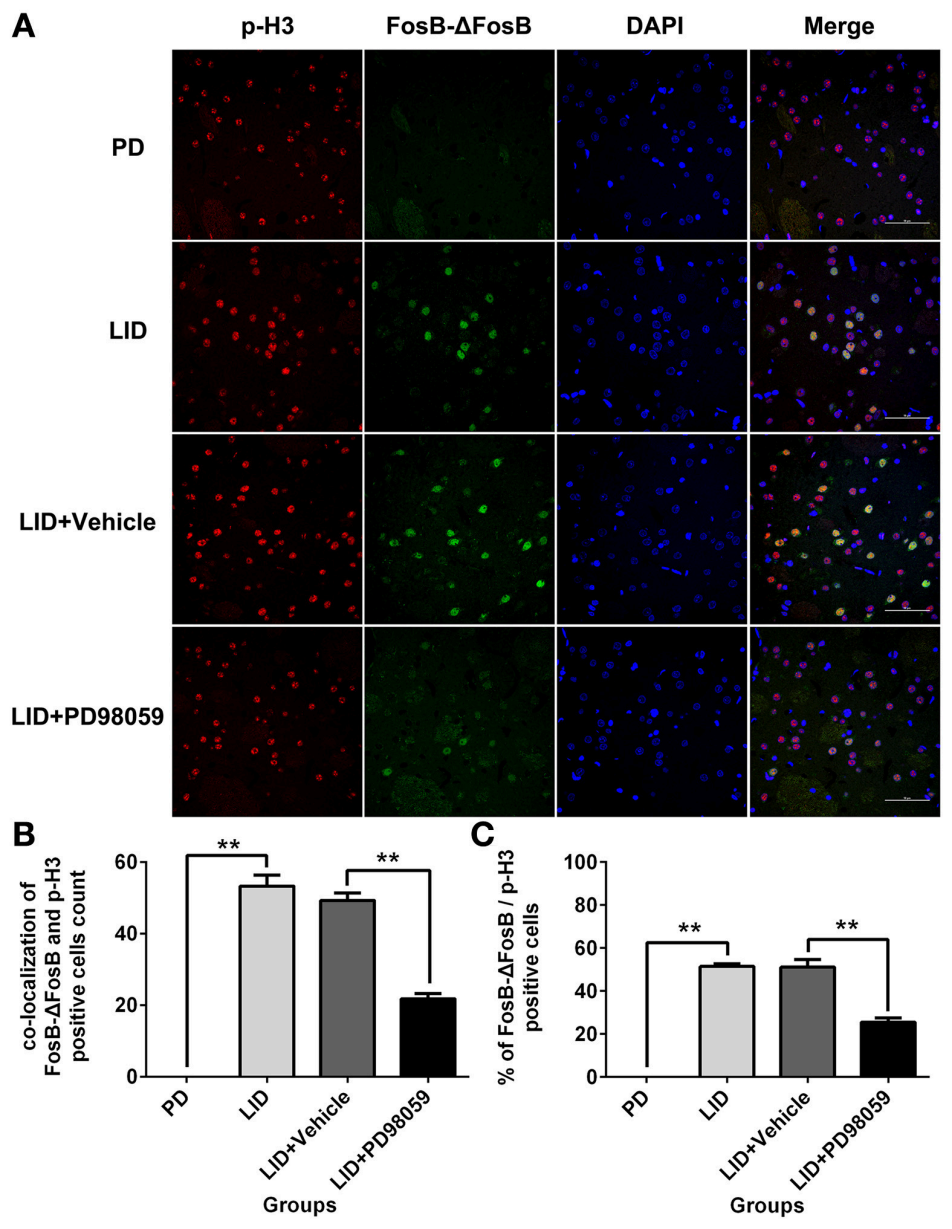
**PD98059 reduces p-H3 and FosB-ΔFosB positively immunoreactive cell counts. (A)** Double immunolabeling images show co-localization between p-H3 and FosB-ΔFosB in the dorsal striatum from each rat group (PD, LID, LID + Vehicle, LID + PD98059). Scale bar is 50 μm. **(B)** Analysis of colocalization of FosB-ΔFosB and p-H3 positive in each group (in an area of 0.05 mm^2^). ^**^p < 0.01 (one-way ANOVA followed by Tukey HSD and LSD *post-hoc* test, *N* = 4/group). **(C)** Percentage of FosB-ΔFosB and p-H3 positive cell count in each group (*N* = 4/group). Error bars represent SEM.

L-DOPA administration induced a significant increase in p-ERK1/2 level in the striatum of unilaterally 6-OHDA-lesioned rats (Figures 3A,B, Supplementary Figure 1). And this effect was prevented by the inhibitor of MEK PD98059 [Figures 3A,B, Supplementary Figure 1; p-ERK1/2 vs. ERK1/2, *F*_(4, 15)_ = 6.244 one-way ANOVA followed by Tukey HSD and LSD *post-hoc* test, *N* = 4/group]. The increase of p-ERK1/2 was accompanied by overexpression of ΔFosB that represents a net increase of ΔFosB (Figures 3A,C, Supplementary Figure 1). PD98059 also decreased ΔFosB levels [Figures 3A,C, Supplementary Figure 1; ΔFosB vs. β-actin, *F*_(4, 15)_ = 15.543 one-way ANOVA followed by Tukey HSD and LSD *post-hoc* test, *N* = 4/group]. Furthermore, PD98059 reduced FosB-ΔFosB immunoreactive neurons in the dorsolateral striatum on the lesioned side [Figures 3D,E; *F*_(4, 15)_ = 989.202, one-way ANOVA followed by Tukey HSD *post-hoc* test, *N* = 4/group]. The number of FosB-ΔFosB immunoreactive neurons of LID + Vehicle and LID + PD98059 groups was 176.5 ± 4.3/section and 90.3 ± 2.9/section, respectively (Figures 3D,E). No difference was found between LID + Vehicle and LID groups. Double immunolabeling images of the two molecules exhibited colocalization between FosB-ΔFosB and p-H3 in rats with dyskinesia (Figure 4A). PD98059 reduced the striatal co-expression of FosB-ΔFosB and p-H3 positive cell count [Figure 4B; co-expression of FosB-ΔFosB and p-H3, *F*_(3, 12)_ = 154.413, one-way ANOVA followed by Tukey HSD and LSD *post-hoc* test, *N* = 4/group]. The quantification of neurons with colocalization in LID + Vehicle group and LID + PD98059 group was 49.25 ± 2.136, 21.75 ± 1.493, respectively (Figure 4B). Quantification demonstrated that about 50 and 25% of striatal p-H3 positive neurons co-expressed with FosB-ΔFosB in LID + Vehicle group and LID + PD98059 group, respectively (Figure 4C). P-H3 negative neurons didn't express FosB-ΔFosB (data not shown).

### MEK inhibitor neutralized changes in gene expression associated with LID

#### Genes associated with LID and regulated by MEK inhibitor

**Figure 5:**
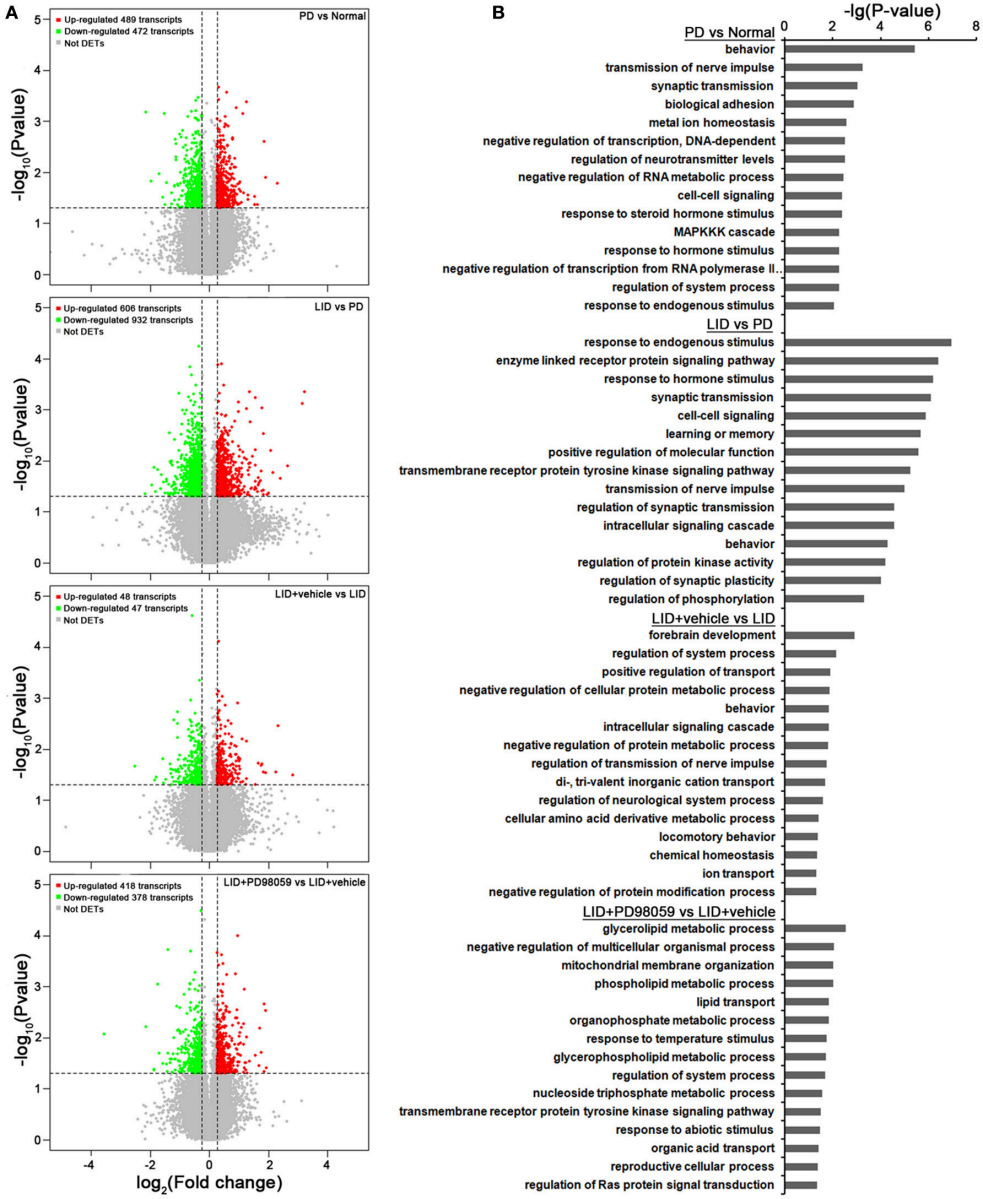
**Microarray analysis. (A)** Gene expression changes induced by each treatment comparing PD vs. Normal, LID vs. PD, LID + Vehicle vs. LID, LID + PD98059 vs. LID + Vehicle (*N* = 3/group; *p* < 0.05, Fold change ≥1.2 or ≤ 0.8333). **(B)** Gene Ontology analysis about the altered genes from the same group comparisons.

Gene expression was compared between the lesioned striatum of rats without L-DOPA treatment (PD group) and that of rats with AIMs after repeated administrations of L-DOPA for 21 days (LID group; ALO AIMs score = 12 at 70 min after L-DOPA). Compared with PD group, LID group exhibits 606 up-regulated transcripts and 932 down-regulated transcripts (*p* < 0.05 and fold change ≥ 1.2 or ≤ 0.8333; Figure 5A). These changes in gene expression are caused by L-DOPA treatment and are mainly related to the following functions, response to endogenous stimulus, enzyme linked receptor protein signaling pathway and several others described in Figure 5B (see also Supplementary Table 2 for details).

In comparison with LID + Vehicle group, LID+PD98059 group exhibits 418 up-regulated transcripts and 378 down-regulated transcripts (*P* < 0.05 and fold change ≥ 1.2 or ≤ 0.8333; Figure 5A). Thus, PD98059 treatment caused large changes in gene expression mainly related to the following functions, negative regulation of multicellular organismal process and several others described in Figure 5B (see also Supplementary Table 4 for details).

Changes in gene regulation between PD and Normal groups and between LID + Vehicle and LID groups were also detected (see Figure 5 and details in Supplementary Tables 1, 3).

**Figure 6:**
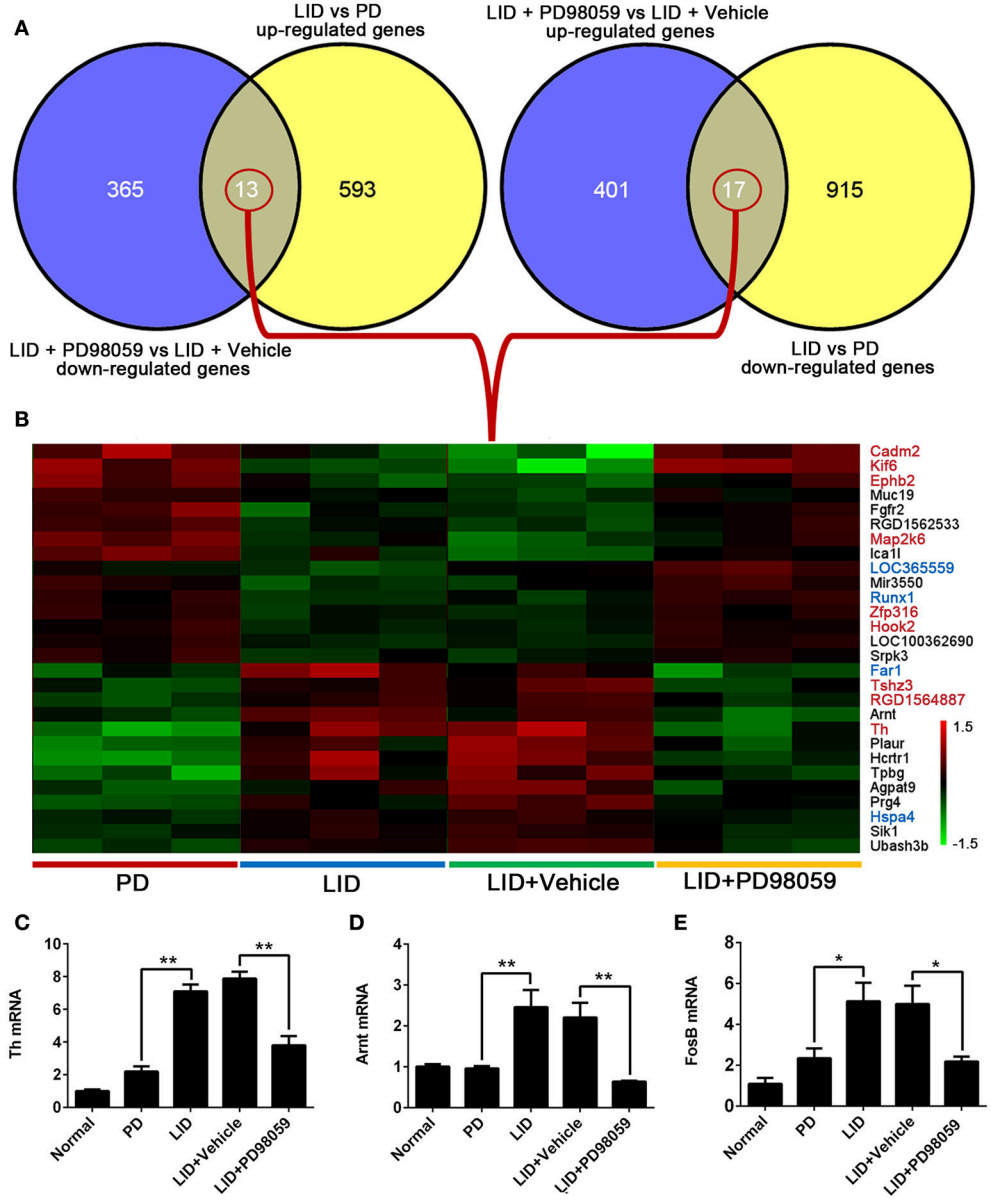
**Genes altered by L-DOPA treatment and responsive to PD98059 treatment. (A)** The number of common genes in opposite trends between LID vs. PD and LID + PD98059 vs. LID + Vehicle (*p* < 0.05, Fold change ≥1.2 or ≤ 0.8333). **(B)** The common genes from the comparison presented in **(A)**. The red and green colors in the heatmap represent up-regulation and down-regulation, respectively. The genes marked with red and blue font in the list represent common genes with red and steel blue module in this figure, respectively. **(C–E)** Changes in *Th, Arnt*, and *FosB* gene expression as detected by quantitative RT-PCR ^*^p < 0.05, ^**^p < 0.01 (one-way ANOVA followed by Tukey HSD and LSD *post-hoc* test, *N* = 3/group).

The results obtained from comparison of groups were further analyzed to identify the genes that were regulated by repetitive L-DOPA, and then changed by PD98059. The analysis showed 13 genes that were up-regulated by L-DOPA and down-regulated by PD98059: *Ubash3b, Sik1, Hspa4, Prg4, Agpat9, Tpbg, Hcrtr1, Plaur, Th, Arnt, RGD1564887, Tshz3, Far1* (Figures 6A,B). Additionally, 17 genes were down-regulated by L-DOPA and then up-regulated by PD98059: *Srpk3, LOC100362690, Hook2, Zfp316, Runx1, Mir3550, LOC365559, Ica1l, Map2k6, RGD1562533, Fgfr2, Muc19, Ephb2, Kif6, Cadm2*, and two unnamed transcripts (Figures 6A,B).

Changes in *Th, Arnt*, and *FosB* gene expression detected with microarray analysis were verified with quantitative RT-PCR (Figures 6C–E). Similar to microarray results, *Th* and *Arnt* were up-regulated by L-DOPA and normalized by PD98059 (*P* < 0.05). Only one of the four probes detecting *FosB* gene (Probe Set ID: 17630237) presented a trend in line with expectations shown in the quantitative RT-PCR result (Figure 6E), namely up-regulated by L-DOPA and down-regulated by PD98059. The gene symbol is set as LOC100360880 in the data, and probe details are shown in the Supplementary Table 5.

#### Identification of PD98059-specific coexpression modules

**Figure 7:**
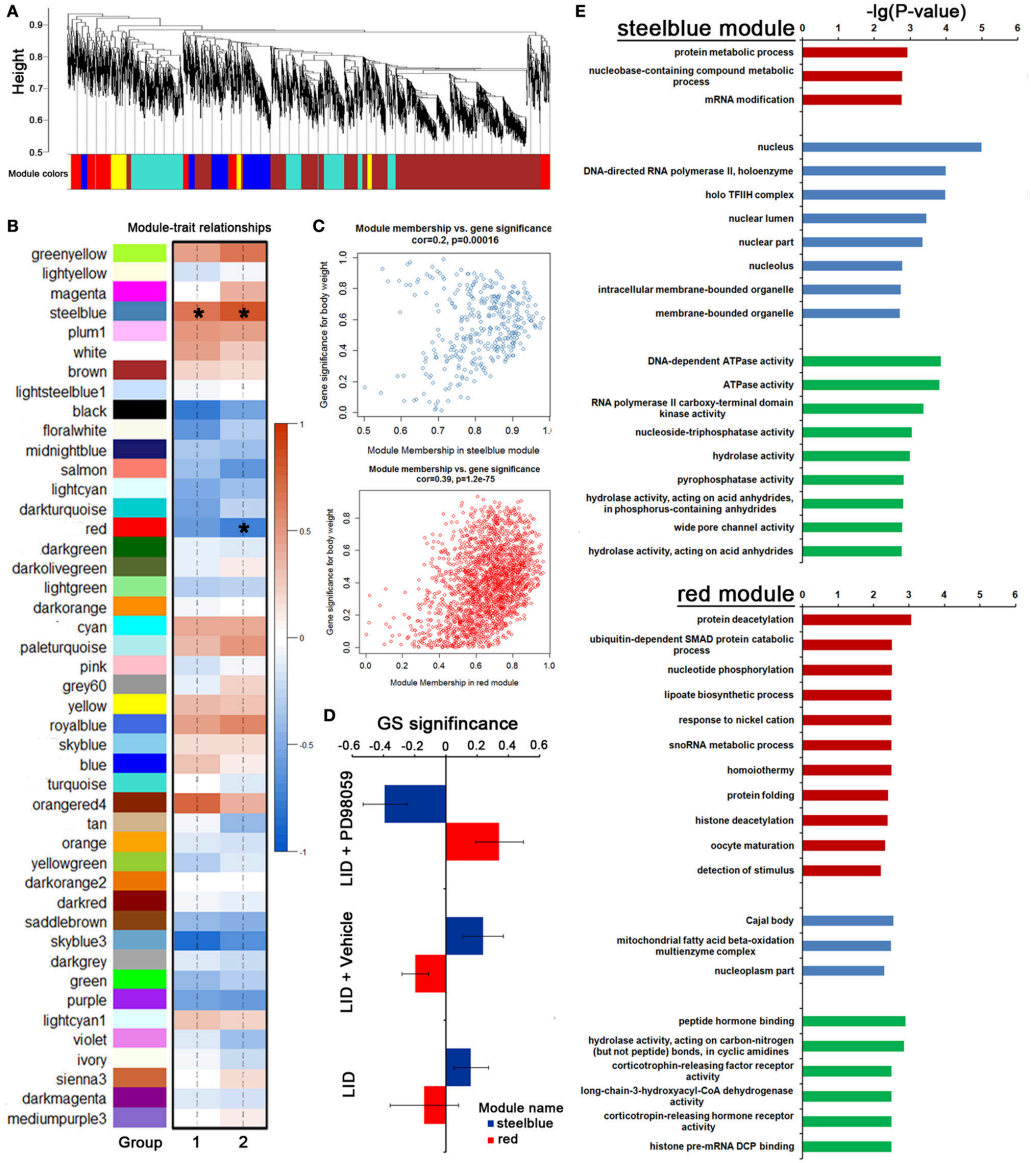
**PD98059-specific co-expression modules across LID+PD98059 group, LID + Vehicle group, and LID groups. (A,B)** All candidates (including protein coding genes and lncRNA) in the dataset (37,177 transcripts in microarray) were hierarchically clustered under unsupervised average linkage and classified into 45 modules labeled by color. **(C)** The module membership (MM) vs. gene significance (GS) plots for steelblue and red module showed that MM and GS are highly correlated (GS *P* < 0.05). **(D)** The steelblue and red module contained candidates negatively and positively correlated to the PD98059 treatment, respectively. **(E)** GO enrichment analysis about transcripts in steel blue and red module (*P* < 0.05).

To further identify a hierarchical network view of co-expressed genes across LID+PD98059 group, LID + Vehicle group and LID group subtypes, we applied WGCNA to a dataset containing 3 specimens in each group. All candidates (including protein coding genes and lncRNA) in this dataset were hierarchically clustered under unsupervised average linkage and classified into 45 modules (Figures 7A,B) labeled by color. Each module was comprised of mutually exclusive co-expressed candidates. Candidates with no distinct module assignment were grouped in a gray module by WGCNA. Two of these modules, containing steelblue and red, were identified using any pre-assigned phenotype (GS *P* < 0.05, Figure 7C). The module membership (MM) vs. gene significance (GS) plots for these modules (Figure 7C) showed that MM and GS are highly correlated, indicating that the candidates most significantly associated with the trait are often also the most important (central) elements of the respective module. Following the unsupervised module generation, individual candidate correlations to a specific treatment were quantified by GS. The average GS of all candidates within each module is summarized in Figure 7D. This analysis unveiled positive or negative correlation of certain modules with PD98059 treatment. The steelblue module contained candidates negatively correlated to the PD98059 treatment (Figure 7D). To validate the robustness of the co-expression network as a specific classifier, it was first applied by unsupervised hierarchical clustering bootstrap analysis to the expression value of each sample in a test dataset from which steelblue module were derived, making it a “PD98059-treatment module,” including protein metabolic process and RNA modification terms with GO enrichment analysis (Figure 7E). Similarly, the red module contained candidates positively correlated to the PD98059 treatment (Figure 7D). And the genes in red module were involved in protein deacetylation and ubiquitin-dependent SMAD protein catabolic process (Figure 7E).

#### Identification and validation of hub networks of coexpressed genes closely related to PD98059 and LID

**Figure 8:**
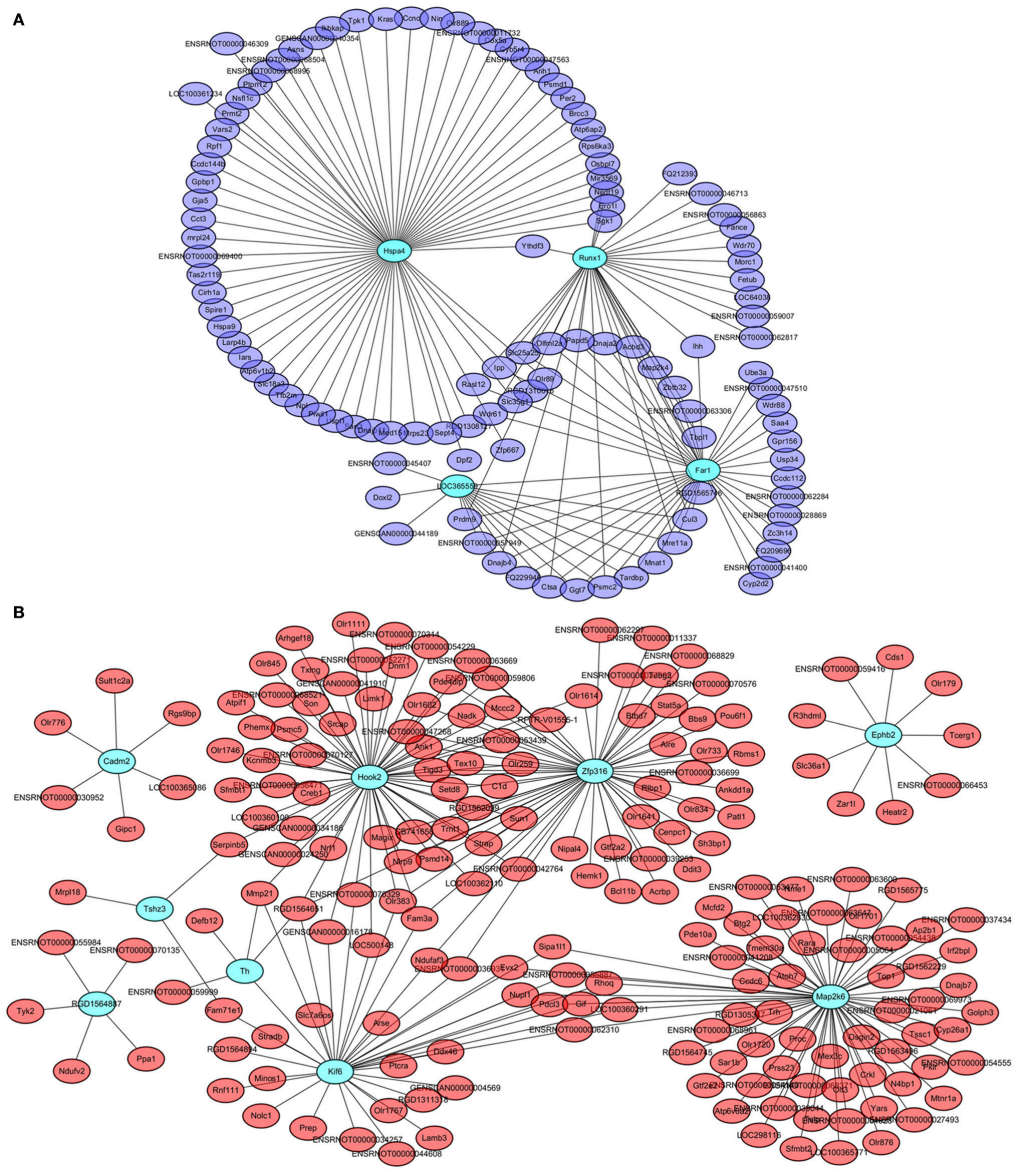
**Hub networks of co-expressed genes closely related to PD98059 and LID. (A)** Hub network of genes correlative with LOC365559, Runx1, Far1, and Hspa4 in the steelblue module. **(B)** Hub network of genes correlative with *Cadm2, Kif6, Ephb2, Map2k6, Zfp316, Hook2, Tshz3, RGD1564887*, and *Th* in the red module.

Four genes in the steelblue module, namely *LOC365559, Runx1, Far1*, and *Hspa4*, and 9 genes in the red module, namely *Cadm2, Kif6, Ephb2, Map2k6, Zfp316, Hook2, Tshz3, RGD1564887*, and *Th*, were also common specific DETs between LID + PD98059 vs. LID + Vehicle and LID vs. PD (red and blue font, respectively; Figure 6B). This overlap suggests that the genes are crucially regulated by PD98059 treatment and LID. We further analyzed these genes in hub networks of co-expressed genes across transcriptome platforms. There were more than 60 DETs in the steelblue module closely related with *Hspa4* (Figure 8A). There were also many DETs in the hub network of the red module related with *Map2k6, Hook2, Zfp316, Kif6*, and *Th* (Figure 8B). The regulation of these genes may thus be related to LID development.

### Effects of MEK inhibitor on the striatal expression of TH and ARNT

**Figure 9:**
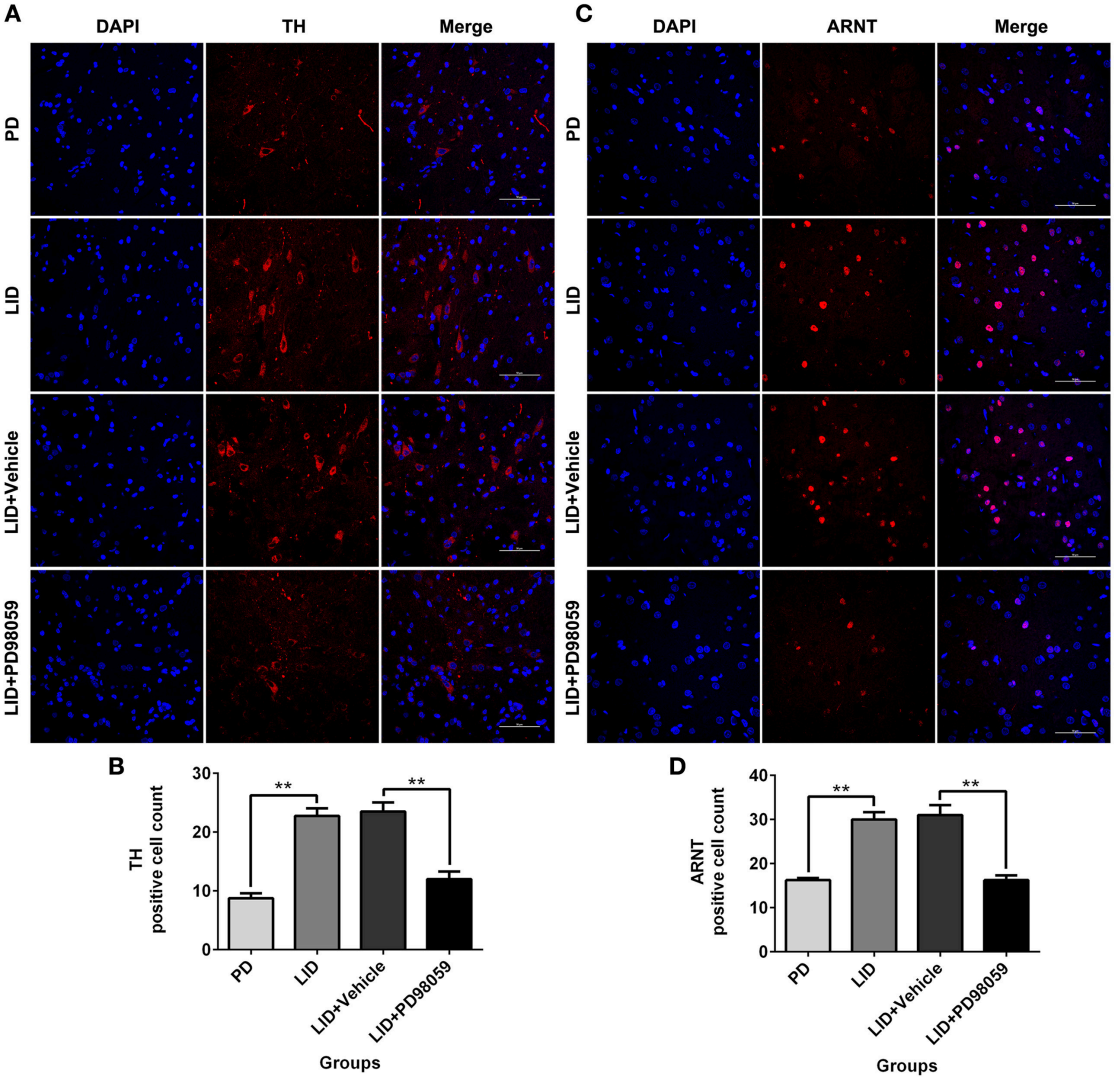
**PD98059 reduces TH and ARNT (HIF-1 β) immunoreactive cells. (A,C)** Examples of immunofluorescent images showing TH and ARNT positive neurons in the dorsolateral striatum. **(B,D)** Analysis of differences in TH and ARNT positive cell counts in each group (an area of 0.05 mm^2^). ^**^p < 0.01 (one-way ANOVA followed by Tukey HSD and LSD *post-hoc* test, *N* = 4/group). Scale bar is 50 μm. Error bars represent SEM.

The expression of TH and ARNT was also analyzed using Immunofluorescence (Figure 8). Chronic L-DOPA administration increased the number of TH and ARNT positive neurons in the striatum of 6-OHDA-lesioned rats (Figure 9). This effect was reversed by the inhibitor of MEK PD98059 [Figure 9; TH, *F*_(3, 12)_ = 34.268; HIF-1 β (ARNT), *F*_(3, 12)_ = 28.696; one-way ANOVA followed by Tukey HSD and LSD *post-hoc* test, *N* = 4/group]. No difference was found between LID + Vehicle and LID groups.

## Discussion

In this study, we showed that lateral ventricle administration of PD98059, a selective MEK inhibitor, could reduce LID in 6-OHDA-lesioned rats. Axial, limb, and orofacial AIMs scores were significantly decreased since the fourth day of PD98059 administration. In addition, rotational behavior had little changes with the addition of the inhibitor indicating that this agent did not neutralize the antiparkinsonian effect of L-DOPA. These findings are in line with previous results showing that SL327, other MEK inhibitor, could significantly reduce LID in 6-OHDA-lesioned mice (Santini et al., 2007) and definitely confirm the key role of ERK1/2 in the development of rodent LID. As classical inhibitors of MEK, PD98059 was widely used in a variety of experiments. It was proved to be highly effective in preventing the phosphorylation of ERK1/2 that could disrupt drug-paired contextual cue memories (Miller and Marshall, 2005). In this study, the increased striatal phosphorylation of ERK1/2 in hemiparkinsonian rats chronically exposed to L-DOPA could also be largely reversed by PD98059.

One of the most significant results of this study is that the antagonistic effect of PD98059 on LID and p-ERK1/2 was accompanied by other specific molecular changes, particularly the reduction of ΔFosB expression. We have confirmed for the first time that inhibiting the over-activation of ERK1/2 could decrease the accumulation of ΔFosB protein in the striatum. The *FosB* gene is ranked in the first place among 28 genes blocked by SL327, other MEK inhibitor with antidyskinetic effects in mice, which is consistent with the present results showing that MEK inhibitor could reduce the transcription of *FosB* (Charbonnier-Beaupel et al., 2015). However, we found in the microarray results that probes for detecting the *FosB* gene (mRNA Accession: NM_001256509) were probably not perfectly designed, although Affymetrix Genechip Rat Gene 2.0 ST was one of the most advanced whole transcriptome gene chip when we started the study. Only one of the four probes (Probe Set ID: 17630237) presented a trend in line with expectations, namely up-regulated by L-DOPA and down-regulated by PD98059. The gene symbol is set as *LOC100360880* in the data and the probe details are shown in the Supplementary Table 5. Furthermore, we carried out real-time quantitative PCR to detect the *FosB* levels in samples. The change pattern among groups confirmed the result of Probe 17630237.

The transcription factor ΔFosB is a recognized hallmark of LID (Andersson et al., 1999; Tekumalla et al., 2001; Cenci, 2002; Cao et al., 2010). Striatal overexpression of ΔFosB could result in abnormal neuronal electrical properties leading not only to dyskinesia but also to an insensitive response to L-DOPA that can be responsive to selective blockade of the expression of this transcription factor (Engeln et al., 2016). The kinetic profile of ΔFosB likely explains the delayed antidyskinetic effect of PD98059 to the fourth day of treatment. This transcription factor possesses unique stability properties compared with all other Fos family members (McClung et al., 2004; Alibhai et al., 2007; Nestler, 2008, 2015), and this is owned to two contributing mechanisms. First, ΔFosB lacks two critical degron domains, which are associated with ubiquitination and degradation in the C-terminus of full-length FosB and all other Fos family proteins. Second, ΔFosB is modified by some protein kinases at the N-terminus, adding further stability to the protein (Ulery et al., 2006; Gajewski et al., 2009; Ulery-Reynolds et al., 2009; Cates et al., 2014). Therefore, ΔFosB proteins tend to accumulate in neurons, and could persist for several weeks after withdrawal of relevant drug exposure. In this study, AIMs were significantly reduced starting several days after initiating MEK inhibitor treatment, and the ΔFosB level was significantly decreased at the end of the experiment. Further studies are necessary to explore the role of certain kinases that mediate ΔFosB catabolism and the contribution of ERK1/2 in the process.

Our microarray data showed that changes in the expression of a considerable number of genes occur with LID development and can be changed by MEK inhibition. Compared with the normal state, there were a total of 961 transcripts changed as a result of dopaminergic lesion. The regulated genes are related directly to synaptic transmission, nerve impulse conduction, behavior and other functions implying that dopamine depletion causes extensive transcriptional alterations. Furthermore, changes in 1,538 transcripts were involved in the development of LID after chronic L-DOPA treatment, and changes in 796 transcripts were associated with PD98059 treatment. The genes regulated with LID and PD98059 widely participate in transmembrane receptor protein tyrosine kinase signaling pathway, synaptic transmission, synaptic plasticity regulation, Ras protein signal transduction regulation, and other relevant functions. Notably, L-DOPA and PD98059 could affect some of the same GO biological processes. The preliminary analysis of GO biological processes, including enzyme linked receptor protein signaling pathway, synaptic transmission, cell-cell signaling, and learning and memory support that these genes, especially *Hspa4, Map2k6, Hook2, Zfp316, Th*, and likely other genes in the hub networks play important roles in the pathophysiology of LID. We focused on the most significant changes in gene expression occurred with PD98059 treatment. According to the in-depth analysis of the data, we found 13 genes that are up-regulated by L-DOPA and could be neutralized by PD98059, and 17 genes that are down-regulated by L-DOPA and could be normalized by PD98059.

It is important to note that among the 13 genes up-regulated by L-DOPA and neutralized by PD98059, one was the abnormal *Th* expression. Several studies have previously demonstrated that striatal Th-positive neurons are correlated with severity of LID and ΔFosB expression in hemiparkinsonian mice (Darmopil et al., 2008; Charbonnier-Beaupel et al., 2015; Keber et al., 2015). Studies have shown that striatal Th neurons may cooperate with serotonergic terminals synthesizing dopamine and producing supraphysiological synaptic DA concentrations, a mechanism thought to contribute to LID (Keber et al., 2015). However, it was also reported that the striatal Th-positive neurons co-expressed with dynorphin and enkephalin, suggesting that they are medium spiny neurons of the direct and indirect striatal output pathways (Darmopil et al., 2008). Clearly, Th colocalization with dynorphin is coherent with our data and the relation to LID because there is sufficient evidence in support of the direct pathway role in LID mechanisms. Instead, Th colocalization with enkephalin is at odds with the specific association of Th expression with LID mechanisms. It is important to consider that the function of newly developed Th expression in striatal neurons in models of PD is still under poorly understood, and it is possible that Th expression serves different functions including a compensatory mechanism to enhance dopamine actions in both striatal pathways. On the other hand, the use of D1 or D2 receptor knock-out mice demonstrated that D1R, but not the D2R is necessary for L-DOPA-induced expression of striatal Th-positive neurons (Espadas et al., 2012). Although, transgenic models of dopamine receptor KO may undergo different gene regulations with L-DOPA treatment, and here we used wild type animals, the present data also support the link of striatal Th expression to LID. In our study, PD98059 significantly neutralized the abnormal Th expression, which suggests a connection between Th and MEK, and possibly p-ERK1/2. Of interest, p-ERK1/2 could indirectly influence Th expression through regulation of the orphan nuclear receptor Nur-related factor 1(NURR1) because putative ERK1/2 phosphorylation sites were found proximal to the N-terminal AF-1 region of NURR1, and NURR1 directly induces transcription of Th gene in midbrain dopamine neurons of the substantia nigra (Jacobsen et al., 2008). However, we have not found a distinct change in the Nurr1 gene with microarray among the studied groups of rats. Therefore, further work is needed to determine whether the connected regulation of p-ERK1/2-NURR1-Th plays a mechanistic role in LID.

Another regulated gene in relation to LID and PD98059 was *Arnt*, whose official full name is aryl hydrocarbon receptor nuclear translocator. ARNT protein is required for activity of the Ah (dioxin) receptor and the ligand-binding subunit to translocate from the cytosol to the nucleus after ligand binding. Then, the complex initiates transcription of genes. Its heterodimer with HIF1A acts as a transcriptional regulator in response to hypoxia (Mannello et al., 2011; Dela Cruz et al., 2014). It was revealed that ARNT, in concert with neuronal PAS domain protein 1 (NPAS1), negatively modulates the expression of *Th* and that this regulation occurs with NPAS1 directly binding on the *Th* promoter (Teh et al., 2007). However, this is conflicting with our data showing that *Arnt* gene expression correlates with *Th* expression changes associated with L-DOPA and PD98059 treatment, upregulation and suppression, respectively. Alternatively, *Th* expression may be induced by other transcription factors, for instance NURR1, and *Arnt* regulation could result from adaptive changes to counteract high *Th* expression.

*Ubash3b* and *Sik1* genes were regulated in parallel to *Th* and *Arnt* in this study. The official full name of *Ubash3b* is ubiquitin associated and SH3 domain containing B. This gene encodes a protein that promotes accumulation of activated target receptors on the cell surface, exhibits tyrosine phosphatase activity, and down-regulates proteins that are dually modified by both protein tyrosine phosphorylation and ubiquitination. One possibility would be that UBASH3B contribute to the accumulation of ΔFosB due to UBASH3B mediated accumulation of activated target receptors. The official full name of *Sik1* is salt-inducible kinase 1. *Sik1* is one of the CREB-target genes. SIK1 induction is thought to act as a negative feedback signal preventing persistent CREB/TORC1-dependent transcription in situations of long-lasting neuronal activity (Hu et al., 2015). It has been shown that Sik1 plays a key role in cocaine addiction (Dietrich et al., 2012), which also shares a mechanism associated with ΔFosB upregulation. *Map2k6* and *Ephb2* are among the 17 genes down-regulated by L-DOPA and up-regulated by PD98059. The official full name of *Map2k6* is mitogen-activated protein kinase kinase 6. MAP2K6 is the activator of p38 MAPK and is involved in synaptic plasticity including cue-induced relapse to heroin seeking, learning and memory (Bolshakov et al., 2000; Fanous et al., 2013). The official full name of *Ephb2* is Eph receptor B2. EphB2 is a member of the EphB family of receptor tyrosine kinases, and could increase synaptic NR1 and NR2B expression, prevent down-regulation of dephosphorylated p38 MAPK and phosphorylated CREB in Aβ1-42 oligomer-treated neurons (Geng et al., 2013). EphB2 is considered a neuroprotective factor for hippocampal neurons with a potential therapeutic role in Alzheimer's disease (Miyamoto et al., 2016). LID is thought to share similar plasticity changes with drug addiction and memory/learning processes. The present study showed that the *Ubash3b, Sik1, Map2k6*, and *Ephb2*, all associated with mechanisms of synaptic plasticity, are regulated by L-DOPA as well as PD98059 treatment.

*Hspa4, Hcrtr1, Fgfr2*, and *Cadm2* are also significantly regulated with L-DOPA and PD98059 treatment. The official full name of *Hspa4* is heat shock protein family A member 4, a member of the heatshock protein (HSP) family 110 (Banduseela et al., 2013). Most HSPs act as chaperones and imperfections in their function can lead to an accumulation of misfolded proteins (Patterson, 2006). HSPA4 has interaction with PD-causing genes, including parkin, DJ-1 and PINK1, although the role of HSPA4 in PD progression and pathogenesis is unclear (van der Merwe et al., 2015). The official full name of *Hcrtr1* is hypocretin receptor 1. HCRTR1 is one of G-protein coupled receptor, and takes part in positive regulation of ERK1/2 cascade and cytosolic calcium ion concentration. The official full name of *Fgfr2* is fibroblast growth factor receptor 2. FGFR2 is an important neurotrophic factor, and may contribute to the development of mesencephalic dopaminergic neurons (Baron et al., 2012). The official full name of *Cadm2* is cell adhesion molecule 2. CADM2 plays an important role for synapse organization, and provides regulated trans-synaptic adhesion (Tanabe et al., 2013). Clearly, a number of other genes could be listed, but these gene regulations need to be investigated in depth to establish their specific relation to behavioral changes and to gain insights into their functional roles.

In conclusion, our results reveal the interplay among p-ERK1/2, ΔFosB, p-H3, TH, ARNT in the mechanisms of LID. Additionally, the series of genes identified in this study are opening new pathways for further insights into the molecular changes associated with LID. Most noticeably are the interrelated regulation of p-ERK1/2-NURR1-TH-ARNT genes and p-ERK1/2-ΔFosB-UBASH3B genes. Further studies of these mechanisms may help identify targets for developing new LID treatments.

## Author contributions

GC collected the data, clustered the literature data, and wrote the paper. SN collaborated in preparation of figures writing the paper. CH and KM collaborated in establishing the model. YX and XC collaborated in writing the paper, supervised the overall work, and revised the paper. ZZ collaborated in referencing the literature and revised the paper. SP advised for data presentation and revised the paper.

### Conflict of interest statement

The authors declare that the research was conducted in the absence of any commercial or financial relationships that could be construed as a potential conflict of interest.

## Supplementary material

Supplementary Table 1 **Gene list of PD vs. normal**.

Supplementary Table 2 **Gene list of LID vs. PD**.

Supplementary Table 3 **Gene list of LID + Vehicle vs. LID**.

Supplementary Table 4 **Gene list of LID + PD98059 vs. LID + Vehicle**.

Supplementary Table 5 **Data of probes detecting FosB/LOC100360880 gene**.

Supplementary Figure 1 **Additional analysis of ERK1/2, p-ERK1/2, and ΔFosB levels**. ERK1/2, p-ERK1/2, and ΔFosB were relatively quantified in the DA-denervated striatum of the fourth rat from each group by Western blotting to include four independent samples in the analysis.
