# Supplementary figures and images for: Antidyskinetic Effects of MEK Inhibitor Are Associated with Multiple Neurochemical Alterations in the Striatum of Hemiparkinsonian Rats
Source: Front Neurosci. 2017 Mar 9;11:112. doi: 10.3389/fnins.2017.00112 (PMC5343040; doi:10.3389/fnins.2017.00112)

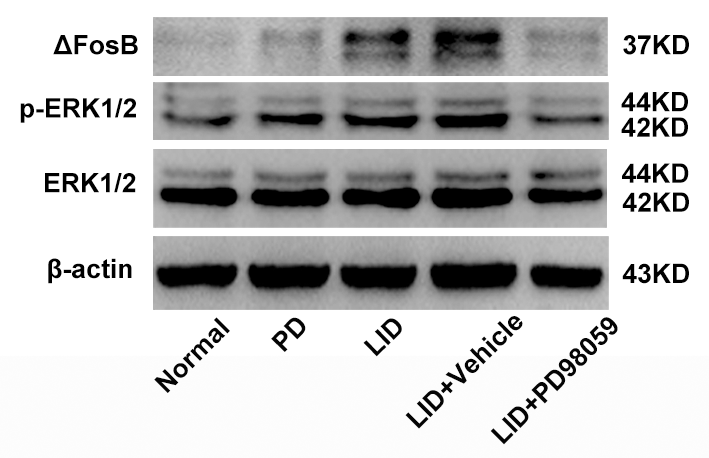

Supplement: Supplementary Figure 1 — Additional analysis of ERK1/2, p-ERK1/2, and ΔFosB levels. ERK1/2, p-ERK1/2, and ΔFosB were relatively quantified in the DA-denervated striatum of the fourth rat from each group by Western blotting to include four independent samples in the analysis. [file Image1.TIF]
